# Education to keep the abdomen relaxed versus contracted during pilates in patients with chronic low back pain: study protocol for a randomised controlled trial

**DOI:** 10.1186/s12891-023-06160-z

**Published:** 2023-01-20

**Authors:** Luciana Crepaldi Lunkes, Milton Apolinário Dias Neto, Lavínia Fernandes Barra, Lívia Resende de Castro, Arthur Sá Ferreira, Ney Meziat-Filho

**Affiliations:** 1 Postgraduate Program in Rehabilitation Sciences, Centro Universitário Augusto Motta (UNISUAM), Rio de Janeiro, RJ Brazil; 2 Physiotherapy Department, Centro Universitário de Lavras (UNILAVRAS), Rua Padre José Poggel, 506, Padre Dehon, Lavras, MG 37203-593 Brazil

**Keywords:** Exercise movement techniques, Chronic pain, Low back pain

## Abstract

**Background:**

Low back pain is a very common symptom frequently characterized as a biopsychosocial problem. This study aims to investigate the effectiveness of education to keep the abdomen relaxed versus contracted during Pilates exercises in patients with primary chronic low back pain.

**Methods:**

Two-group randomised controlled trial with allocation of parallel groups and intention-to-treat-analysis. This study will be conducted in Lavras, MG, Brazil. A total of 152 participants will be randomised into two groups that will be treated with Pilates exercises for 12 weeks (twice a week for 60 minutes). Recruitment began in May 2022. The control group will receive guidance on the specific activation of the center of strength (the powerhouse), while the experimental group will receive guidance to perform the exercises in a relaxed and smooth way. Primary outcomes will be pain intensity (Numeric Pain Rating Scale) and disability (Rolland-Morris Questionnaire) 12 weeks post randomisation. Secondary outcomes will be global improvement (Perception of Global Effect Scale) and specific functionality (Patient-specific Functional Scale). The outcomes will be analyzed using repeated-measure linear mixed models. The assessors were not considered blinded because the participants were not blinded, and outcomes were self-reported.

**Discussion:**

The findings of this study will help in clinical decision-making concerning the need to demand abdominal contraction during the exercises, understanding if it’s a fundamental component for the effectiveness of the Pilates method for this population.

**Trial registration:**

This trial was prospectively registered in the Clinical Trials (NCT05336500) in April 2022.

**Supplementary Information:**

The online version contains supplementary material available at 10.1186/s12891-023-06160-z.

## Background

Low back pain is a very common symptom and the causes of its onset and its specific sources of nociception are rarely identified [1]. It is characterized often as a biopsychosocial problem associated with the experience of pain and disability, covering a range of factors that include biophysical, psychological, and social dimensions. Low back pain impairs social function and participation, in addition to personal financial prosperity, since additional costs are associated with chronic conditions [2]. There is a need to reduce unnecessary and even harmful health care, and most importantly, to promote an active lifestyle and adopting healthier habits [3].

The Pilates method is an important component in the treatment of primary chronic low back pain [4–6]. Hayden et al. (2021) conducted a systematic review evaluating the impact of exercise treatment on pain and functional limitations in adults with chronic nonspecific low back pain. They emphasized Pilates as one of several effective methods when compared to no treatment, placebo or usual care [7]. In patients with chronic low back pain, the beneficial effects of Pilates have been observed within 12 weeks, mainly in reducing pain and improving function. Sessions are recommended to last around 60 minutes, with a frequency of two and three times a week, supervised by certified physical therapists [8]. It is important that the exercises include isometric strengthening, global stretching, breathing, and proper positioning of the spine, promoting greater body awareness [9].

Pilates is an effective exercise method for rehabilitation of musculoskeletal disorders, as its principles are based on the activation of local muscles [10]. Specifically in chronic low back pain, clinical trials conducted using exercise protocols based on the principles of the method demonstrate its effectiveness [11–15]. In this sense, motor control of the lumbar spine and maintenance of adequate body posture during the execution of each Pilates exercise is relevant [16].

The real importance of activating muscles such as transversus abdominis and multifidus in chronic musculoskeletal pain conditions is still debatable [17, 18]. Contrary to the fact that muscular components of the trunk influence body stabilization, voluntary activation of the abdominal bracing can limit movements and increase mechanical stress, resulting in greater energy consumption [18, 19]. In patients with chronic low back pain, the transversus abdominis muscle appears to show no delay during activation [17]. Furthermore, multifidus activity can be modified independently of abdominal bracing activation [18]. Individuals with chronic low back pain exhibit higher global trunk muscle activity (multifidus, erector spinae, external oblique, and rectus abdominus )[20]. Interestingly, symptomatic individuals have increased erector spinae muscle activity (longissimus, iliocostalis, and multifidus) during the performance of functional tasks, contradicting the idea of stimulating the muscle activity [21]. Interestingly, patients with chronic low back pain have increased erector spinae muscle activity (longissimus, iliocostalis, and multifidus) during the performance of functional tasks, contradicting the idea of stimulating the muscle activity on these patients [21].

Consequently, a question arises about the need to encourage abdominal muscles contraction instead of relaxation during the performance of Pilates method exercises for the treatment of chronic low back pain. Therefore, the primary objective of this study is to investigate the effectiveness of education to keep the abdomen relaxed compared to contracted during Pilates exercises in reducing pain and disability in patients with primary chronic low back pain 12 weeks after randomisation.

## Methods and design

### Design and setting

**Fig. 1:**
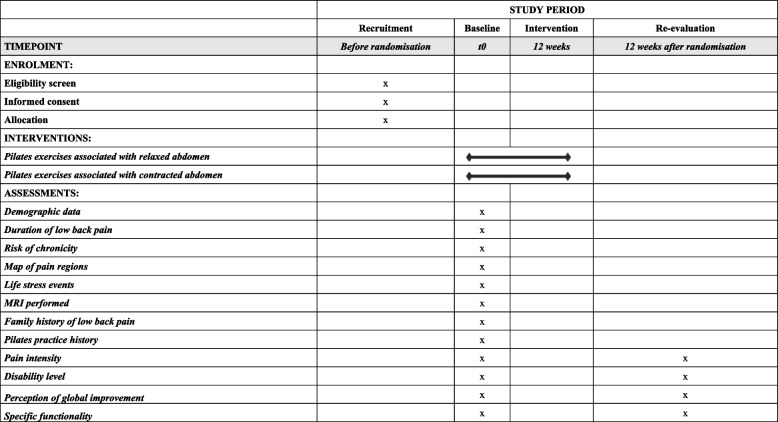
Content for the schedule of enrolment, interventions, and assessments (SPIRIT 2013)

**Fig. 2:**
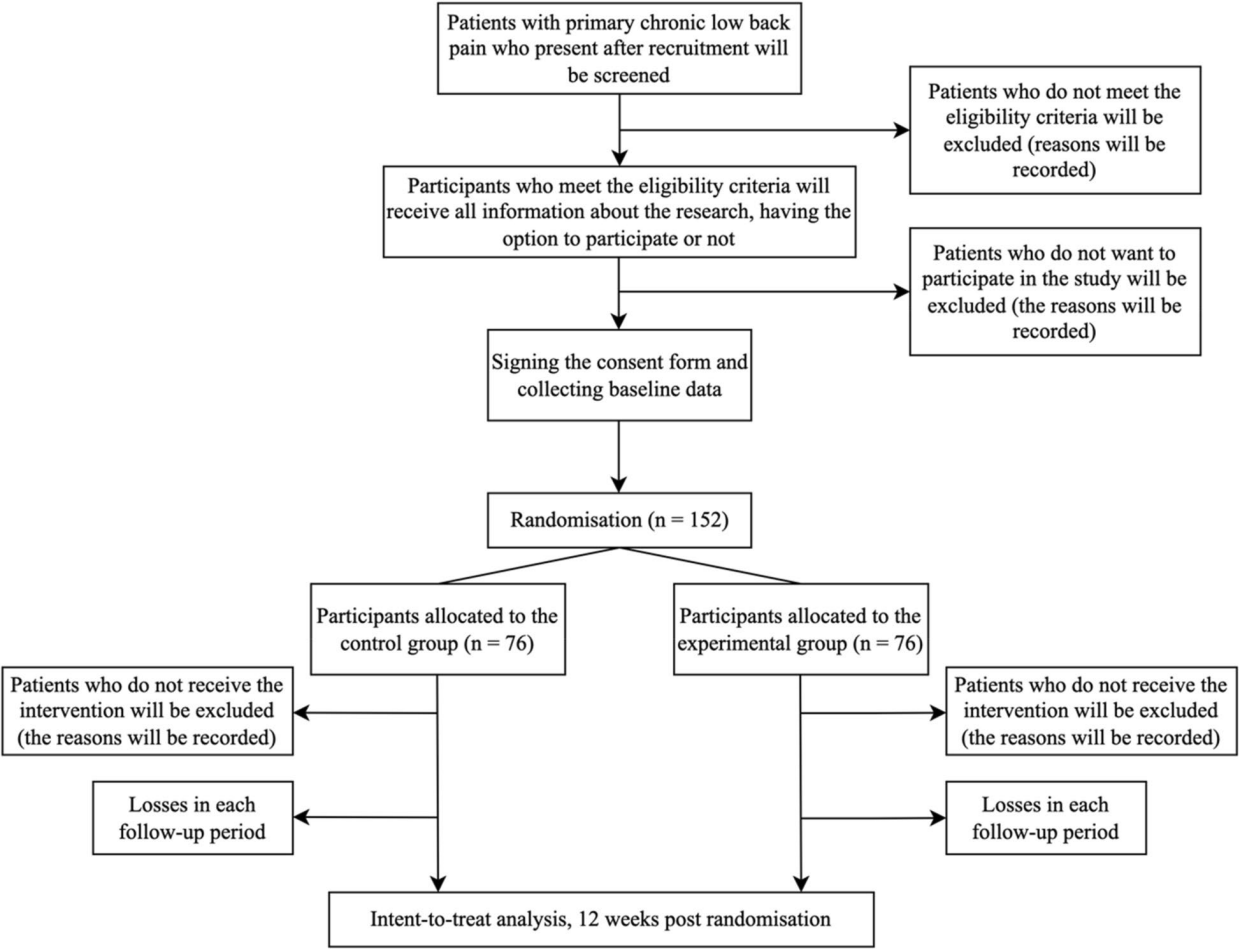
Study flowchart

The protocol was written according to the SPIRIT statement (Fig. 1), increasing its transparency and completeness [22]. The findings will be reported following the CONSORT statement [23] and the TIDieR checklist [24]. This study is a research protocol for a two-group randomised controlled trial with parallel-group allocation and intention-to-treat analysis (Fig. 2). The study will be conducted in Lavras, MG (Brazil).

### Recruitment and participants

We will screen patients of both sexes with primary chronic low back pain with symptoms for at least 12 weeks and who have not had an episode of worsening pain in the last 6 weeks (Table 1). Initially, participants will be evaluated by a physical therapist who is blinded to the allocation of intervention groups, confirming their eligibility according to the inclusion and exclusion criteria (Table 2). This screening will involve the survey of a brief history and the application of a questionnaire related to disability, as it characterizes one of the inclusion criteria. Eligible patients will be informed about the objectives and procedures of the study. They will be informed that there are two active interventions, but it is not yet known which one is superior. Eligible patients that agree to participate in the study will sign a print informed consent form before enrollment. The study started in May 2022, and the recruitment rate is 10 patients per month on average.

**Table 1:** Execution schedule – recruitment, intervention, and reassessment (SPIRIT schedule of enrollment, interventions, and assessments)

	Recruitment	Baseline	Intervention period	Re-evaluation
			12 weeks	12 weeks after intervention
**Before randomisation**				
Eligibility Criteria	x			
Selection	x			
Informed consent	x			
**Primary outcomes**				
Pain intensity		x		x
Disability level		x		x
**Secondary outcomes**				
Perception of global improvement		x		x
pecific functionality		x		x
**Characteristics of participants**				
Demographic data		x		
Duration of low back pain		x		
Risk of chronicity		x		
Map of pain regions		x		
Life stress events		x		
MRI performed		x		
Family history of low back pain		x		
Pilates practice history		x		
Pilates exercises associated with relaxed abdomen			x	
Pilates exercises associated with contracted abdomen			x

**Table 2:** Inclusion and exclusion criteria for research participants

Inclusion criteria	Exclusion criteria
Aged from 18 years old; Low back pain for more than 3 months; Disability level ≥ 4 points on the Roland-Morris Disability Questionnaire (RMDQ); Ability to move independently with or without assistance; Ability to understand Portuguese enough to be able to fill in the questionnaires.	The main area of pain other than the lumbar spine (eg, nerve root compression or herniated disc with radicular pain, lateral or central stenosis); Surgery performed on the lumbar spine; Surgery performed on the lower limbs, or abdominal region less than 6 months ago; Performing an invasive procedure for pain relief (eg, epidural injection, rhizotomy) in the last 3 months; Diagnosis of inflammatory rheumatologic diseases, progressive neurological disease, or viral diseases as the primary cause of pain; Scoliosis as the primary cause of pain; Unstable heart conditions; Presence of red flags such as malignancy/cancer, acute trauma (less than 6 months), infection, spinal cord/cauda equina compression.

### Treatment allocation and randomisation

Initially, a random sequence in blocks will be generated through a computer program by a researcher not involved in data collection. The random codes for treatment will be placed in opaque, sealed envelopes and sequentially numbered, thus assuring the secret allocation of individuals to the study groups. After the initial assessment, inclusion in the study, and baseline data collection, a researcher will open the envelopes and refer the participant to the physical therapist responsible for the respective group of allocation. The patient will start treatment in the same week, with groups of up to 6 patients for each treatment.

### Intervention

Patients included in the study will be randomised to receive Pilates method exercises associated with education to keep the abdomen relaxed (KAR) or contracted (KAC). A certified physiotherapist in the Pilates method with at least 10 years of clinical practice has trained two other physiotherapists, both certified in the Pilates method (100 hours of training), for the exercise protocol. The training performed for the exercise protocol of both physiotherapists was the same. The training for the instruction type used in each intervention group was carried out individually. In addition, a pilot study was performed with 16 volunteers for 3 months.

Pilates method is characterized by exercises that involve the body and mind [25]. Its principles include 1. Centralization, based on the activation of the core or powerhouse muscles (transversus abdominis, diaphragm, obliques, multifidus, and pelvic floor) during exercises, as they are involved in the stabilization of the lumbopelvic complex; 2. Concentration, focusing attention on proper exercise performance; 3. Control, the exercise is performed with concentration, movement, and posture control; 4. Precision, which refers to paying attention to the quality of the exercise technique; 5. Breathing, the exercises are performed in the rhythm of breathing, promoting the activation of the deep muscles of the trunk; and 6. Flow: smoothness during exercise [26–28].

Based on validated protocol by Brito da Silva et al. (2018), nine exercises will be proposed: Spine stretch, The spine twist, The hundred, The one leg circle, The plank, Leg pull front, Swimming, Rocking, and Swan (Additional file 1: Appendix 1) [29]. All exercises will be performed in a single series, with 10 repetitions, previously demonstrated by the physical therapist. Aiming that the protocol can be executed in any circumstances, equipment and materials will not be used. The exercises will always be performed in groups of a maximum of six patients, however the progression in each exercise will be individualized. The sessions will last 60 minutes, twice a week until the 12 weeks are completed, without prescription of home exercises. Sessions will be conducted in different environments and without any contact between therapists and participants.

In the KAC group (control), patients will receive instructions about breathing and its performance along with muscle action. Patients will receive verbal instructions about the performance of each exercise. During execution, postures will be corrected as needed. All patients in the KAC group will be instructed about the performance of exercises based on specific activation of the musculature (transversus abdominis, diaphragm, obliques, multifidus, and pelvic floor). This characterizes the contraction of the center of strength (core or powerhouse), conceptualized by stabilization depth and activation of abdominal bracing. During the execution of the exercises, the patients will receive stimuli through verbal commands, which will be continuously reinforced, so that the contraction of the muscles is performed throughout the protocol. In the KAR group (experimental), patients will be instructed to perform the exercises in a relaxed and smooth way, keeping only breathing and concentration during their execution. Also, at no time will the participants be instructed to perform abdominal muscle contraction or activation of the center of strength.

The assessment of treatment fidelity will be performed by in vivo observations as well as video recording during the pilot study and along the trial. Therefore, both groups will be monitored, ensuring adequate adherence to the treatment that will be performed as planned.

### Characteristics of participants

Baseline characteristics will include sociodemographic information, symptoms duration, number of pain areas, family history of low back pain, magnetic resonance imaging (MRI) performed, chronicity risk (Örebro Musculoskeletal Pain Questionnaire [OMPQ]), history of Pilates method practice, and life stress events (Table 3). Baseline characteristics (e.g. history of Pilates method practice) may be analyzed as potential predictors or moderators of outcomes.

**Table 3:** Characteristics of participants

1) Sociodemographic data, including age and sex, will be obtained; 2) Duration of low back pain (in months) will be obtained through the question: “How long have you had low back pain?”; 3) Risk of chronicity, obtained through the 10 items of the short version of the Örebro Musculoskeletal Pain Questionnaire (OMPQ)[30]; 4) Map of pain regions, where the participant will mark the numbers that correspond to the pain site; 5) Life stress events assessed through closed questions, with dichotomous answers (yes and no), covering the following aspects: serious illness that resulted in withdrawal from usual activities; hospital admission due to illness or accident; death of a close relative; severe financial problems; forced change of housing; separation/divorce; Physical aggression; and assault/robbery; 6) MRI performed, where the following question will be asked: “Have you ever performed a magnetic resonance imaging (MRI) for the lumbar spine?” Answer options: yes or no; 7) Family history of low back pain, where the following question will be asked: “Do you have anyone in the family who suffers from low back pain?” Answer options: yes or no; 8) History of Pilates method practice, where the following question will be asked: “Have you ever practiced Pilates?” Answer options: yes or no.	

### Outcome measures

Co-primary outcomes will be pain intensity (Numerical Pain Scale) and disability (Roland-Morris Disability Questionnaire) related to primary chronic low back pain assessed 12 weeks after randomisation, obtained through self-report. Secondary outcomes will be global improvement (Perception of Global Effect Scale) and specific functionality (Patient-specific Functional Scale) assessed 12 weeks after randomisation (Table 4).

**Table 4:** Primary and secondary outcomes

Outcome	Measurement
1st) Pain intensity	The Brazilian version of the 11-point Numerical Pain Scale (NPS) [22, 31], which varies between 0 and 10 points, where 0 is “no pain” and 10 is “the worst pain imaginable” where individuals should report pain intensity based on the last 7 days;
2nd) Disability	The Brazilian version of the Roland-Morris Disability Questionnaire (RMDQ) [32], which has 24 statements involving activities and pain situations, to which the patient is instructed to answer “yes” only to those described in the assessment day, and each answer corresponds to one point;
3rd) Perception of global improvement	Perception of Global Effect Scale (PGES), 11 points ranging from −5 (“much worse”), 0 (“no change”) to + 5 (completely recovered) [22]. For all measures of perceived global effect participants will be asked: “Compared to the start of your first episode, how do you describe your low back pain in the past few days?”. A highly positive score indicates greater recovery, while a negative score indicates worsening of symptoms;
4th) Specific functionality	Patient-specific functional scale (PSFS) [22], which consists of an interview in which the patient chooses three important activities whose execution is difficult or impossible due to low back pain. For each activity, a score of difficulty ranges from 0 (unable to perform) to 10 (able to perform at the same level as before the problem).

### Blinding

It was not possible to blind the participants and the treating clinicians. The assessors were not considered blinded because the participants were not blinded, and outcomes were self-reported. However, to guarantee that the treatment expectation was evenly balanced between the groups and decrease measurement bias, the participants will not know the study hypothesis, and the assessors will not know the participant’s intervention group. The statistician will be blinded to the group allocation.

### Statistical analysis

The analysis of the data obtained during the research will be carried out by a statistical professional who will have access to the coded data and were based on intention-to-treat principles. Missing data will be assumed to be missing completely at random. Multiple imputation will be used to account for these missing data [33]. Missing values in outcome variables will be estimated using multiple imputation by chained equations after 50 replicated imputed data sets. Variables included in the multiple imputation process will include (1) group factor, (2) time factor, and (3) the respective outcome variable. Descriptive statistics will be used to present the characteristics of participants in the two treatment groups. Two-sided *P* values of less than 0.05 will be considered to indicate statistical evidence of significance. The outcomes pain intensity and disability will be analyzed using repeated-measure linear mixed models (participants and time as random factors) that included all the scores that will be reported after randomisation with the baseline scores and treatment clusters as covariates. Adjusted mean differences will be tested at 12 weeks after randomisation. Multiple comparisons will be performed using the Tukey test with *P* values adjusted using the Holm procedure. The baseline variables will be evaluated as predictors and moderators of treatment effects including terms and interaction models. Effect sizes for primary and secondary outcomes will be calculated as Cohen’s d from estimated marginal means and standard error estimates from the primary adjusted analysis. Effect sizes will be interpreted according to Cohen’s criteria (small ≤0.2; moderate = 0.5; large ≥0.8) [34]. All analyses will be performed using the RStudio version 0.99.486.

### Sample size estimation

The sample size required for this study will be 152 (76 per group) to detect a mean difference of 4.0 points for disability measured by RMDQ and 1.0 for pain intensity measured by NPRS between the two intervention arms, assuming a standard deviation of 4.9 for disability and 2.0 for pain, with an alpha of 5%, power of 80% and a possible loss to follow-up of 15 % [35–37].

## Discussion

The results of this study will contribute to the understanding of the effectiveness of education to keep the abdomen relaxed versus contracted during Pilates method exercises in the treatment of primary chronic low back pain. Pilates is commonly used as an effective treatment for low back pain [38]. In Brazil, the Pilates method is widely disseminated, including practitioners of different profiles and age groups. It is used by physiotherapists for the prevention, recovery, and maintaining the overall functioning of the body.

There are no comparisons between these different strategies during the practice of Pilates method exercises – education based on a contraction versus relaxation of the abdomen. In this sense, aiming to understand the value of each component and increase the effect size of Pilates treatment, the result of a randomised controlled clinical trial of high methodological quality will help physical therapists in the clinical decision-making process.

There will be no objective measure of abdominal muscles activation in our study. However, regardless of whether the participant is able to activate the muscles as requested by the physiotherapist, the instruction for contracting the abdominal itself is an instruction for protecting the lower back during the exercises as opposed to the experimental group.

The sample size of this study will be sufficient to detect the relevant effects of different protocols conducted with a low risk of bias. Increasing the relevance of the study, the exercise protocol will be designed to reproduce the same format in which they would be conducted in the clinical practice by a certified physical therapist. The conclusions of this study may contribute to the existing literature for the treatment of patients with primary chronic low back pain with Pilates.

## Trial registration in a clinical trials database

This study was prospectively registered in the Clinical Trials (Registration number: NCT05336500) and approved by the Human Research Ethics Committee (Plataforma Brasil CAAE 25669519.4.0000.5116). Recruitment begins in May 2022 and is expected to continue until March 2023.

## Supplementary Information

**Additional file 1: Appendix 1.** Exercise protocol based on the Pilates method.

## Data Availability

All data measured and/or manipulated during the study will be available by the corresponding author on reasonable request. Patients will be accompanied by the physiotherapist responsible for their treatment. All possible adverse effects or events during the intervention will be recorded daily. An auxiliary researcher will act as data coordinator at baseline and during reassessments, being responsible for all the processes involved until the end of the research (completion of tables and data processing). All documents will be stored in a safe place, being accessible only to the researchers involved. Electronic data will be protected on a computer with a restricted password and access, and only authorized researchers will be able to handle them. All names or personally identifiable information will be encoded. The statistician who will do the final analysis will receive the coded data.

## Abbreviations

CAPES: Coordenação de Aperfeiçoamento de Pessoal de Nível Superior
FAPERJ: Fundação Carlos Chagas Filho de Amparo à Pesquisa do Estado do Rio de Janeiro
KAC: Education to keep the abdomen contracted
KAR: Education to keep the abdomen relaxed
NPS: Numerical Pain Scale
OMPQ: Örebro Musculoskeletal Pain Questionnaire
PGES: Perception of Global Effect Scale
PSFS: Patient-specific functional scale
RMDQ: Roland-Morris Disability Questionnaire
