# Correction: Education to keep the abdomen relaxed versus contracted during pilates in patients with chronic low back pain: study protocol for a randomised controlled trial

**DOI:** 10.1186/s12891-023-06224-0

**Published:** 2023-02-13

**Authors:** Luciana Crepaldi Lunkes, Milton Apolinário Dias Neto, Lavínia Fernandes Barra, Lívia Resende de Castro, Arthur Sá Ferreira, Ney Meziat-Filho

**Affiliations:** 1 Postgraduate Program in Rehabilitation Sciences, Centro Universitário Augusto Motta (UNISUAM), Rio de Janeiro, RJ Brazil; 2 Physiotherapy Department, Centro Universitário de Lavras (UNILAVRAS), Rua Padre José Poggel, 506, Padre Dehon, Lavras, MG 37203-593 Brazil

**Correction: BMC Musculoskelet Disord 24, 49 (2023)**

10.1186/s12891-023-06160-z

Following publication of the original article [1], the authors reported that one reference citation in Table 4 (ref. [22]) is incorrect. It should be "(Costa LOP, Maher CG, Latimer J, Ferreira PH, Ferreira ML, Pozzi GC, et al. Clinimetric testing of three self-report outcome measures for low back pain patients in Brazil: which one is the best? Spine (Phila Pa 1976). 2008;33:2459–63 Available from: http://ovidsp.ovid.com/ovidweb.cgi?T=JS&PAGE=reference&D=emed8&NEWS=N&AN=2009254927)” instead of Chan et al. 2013.

Furthermore, the word "Specific" in Secondary outcomes is without the "S" in Table 1.

The original article [1] has been updated.
