# Supplementary material for: Education to keep the abdomen relaxed versus contracted during pilates in patients with chronic low back pain: study protocol for a randomised controlled trial
Source: BMC Musculoskelet Disord. 2023 Jan 20;24:49. doi: 10.1186/s12891-023-06160-z (PMC9854226; doi:10.1186/s12891-023-06160-z)
Supplement: Supplementary file 1 — Additional file 1: Appendix 1. Exercise protocol based on the Pilates method. [file 12891_2023_6160_MOESM1_ESM.docx]

**Supplementary material**

Appendix 1. Exercise protocol based on the Pilates method.

Participants will be instructed to breathe correctly through deep and complete inspiration and expiration (chest inspiration, upper chest expiration, lower chest expiration, abdominal expiration).

All exercises will be performed in a single series, with 10 repetitions, previously demonstrated by the physical therapist:

1. Spine Stretch: Sitting on the ischia, maintaining the alignment of the physiological curves of the spine, with knees in extension and hips in abduction. Hands positioned in front of the body (shoulders flexed at 90 degrees, elbows extended) starting the movement from the cervical vertebrae, curling up the spine, and returning to the initial posture. Progression: hips in maximum abduction with ankle in dorsiflexion, with hands over de toes.

2. The Spine Twist: Sitting on the ischia, maintaining the alignment of the physiological curvatures of the spine, with knees in extension and hips in abduction. Shoulders aligned at 90 degrees of flexion and abduction, elbows extended. One hand positioned towards the opposite foot, returning to the initial posture, followed by the other hand. Progression: hips in maximum abduction, increasing trunk rotation.

3. The Hundred: In dorsal decubitus, hips and knees flexed at 90 degrees, ankles in plantar flexion. The arms rest at the side of the body, with elbows extended, and palms supported. Move the arms in the air quickly, in sync with the breath, while performing cervical flexion, removing the scapulae from the ground. Progression: hips and knees extended.

4. The One leg circle: In dorsal decubitus, lower limbs in extension, ankles in plantar flexion. The arms rest at the side of the body, with elbows extended, and palms supported. Perform rotational movements with one of the legs, keeping the iliac spines facing upwards, followed by the other leg. Progression: cervical flexion, removing the scapulae from the ground.

5. The Plank: Side plank – in lateral decubitus, perform weight-bearing on the forearm at an angle of 90 degrees. Knees remain flexed at 90 degrees, hips in neutral position (without support), maintaining isometry. Opposite armrests at the side of the body. Progression: perform weight-bearing on the arm extended.

6. Leg Pull Front: Four base position (hips and knees flexed at 90 degrees, supported on the floor; shoulders and elbows flexed at 90 degrees, with the palms hands resting on the floor). Extend one leg concurrently with the contralateral arm, maintaining the alignment of all segments, including the curvatures of the spine. Return to the starting position and continue with the opposite limbs. Progression: elbows extended.

7. Swimming: Prone position, knees, and shoulders extended, arms stretched in front of the body. Alternately, dissociate upper and lower limbs, performing a movement similar to swimming. While elevating an upper limb, extend the contralateral hip. Progression: spinal extension with hyperextension of the contralateral hip.

8. Rocking: Prone position, with knees in maximum flexion. Hands hold the feet, keeping the elbows in flexion. Perform elbow extension, seeking knee extension. Progression: spinal hyperextended without oscilating.

9. Swan: Prone position, with hands resting on the floor towards or above the shoulders. Raise the trunk, pushing the ground with the hands, keeping the pelvis in contact with the support surface. Progression: brong the arms closer to the body.
